# The Scientific Aspect of Brown-Sequard’s Discovery

**Published:** 1889-09

**Authors:** 


F. E. DANIEL, M. D., EDITOR AND PUBLISHER.

THIS JOURNAL, by unanimous vote of the members, was made the Official Organ of the Austin District Medical Society.

COLLABORATORS:

*Dr. R. M. Swearingen, Austin.*
*Prof. B. E. Hadra, M. D., Galveston.*
*Prof. Geo. Cupples, M. D., San Antonio.*
*Dr. T. C. Osborn, Cleburne, Texas.*
*Dr E. J. Doering, Chicago.*
*Dr. E. J. Beall, Fort Worth.*
*Dr Odo Betz, Germany.*
*Dr. J. W. McLaughlin, Austin.*
*Dr. T. J. Tyner, Austin.*
*Prof. J. F. Y. Paine, M. D., Galveston.*
*Dr. R. H. L. Bibb, Mexico.*
*Dr. T. J. Bennett. Austin.*
*Dr. Bat Smith, Wharton.*
*Dr. E. Meierhof, New York.*
*Prof. W. B. Rogers, M. D., Memphis, Tenn.*

### THE SCIENTIFIC ASPECT OF BROWN-SEQUARD'S DISCOVERY (?)

### A GRAY HORSE OF ANOTHER COLOR.

In our last issue we said, "To us, there is something indescribably ludicrous in the idea, or association of ideas, that a general tonic or nerve stimulant was to be found in the testicles of a young animal."

Now we wish we hadn't said it. There are more strange things in chemistry than ever were dreamed of in our philosophy, Horatio,—and we have "put our foot in it." Well, there is consolation in the reflection that wiser men than we, to-wit, the eurdite editor of the Medical and Surgical Reporter, and others, who have ridiculed the idea, are in the same boat, and if we are laughed at, they will be.

It transpires that Parke, Davis & Co. have, with that intelligent zeal in the cause of new remedies, which for years has characterized them, sought, found and isolated the active principle of the "juice" prepared according to Brown-Sequard's formula, and they recognize it as "spermine"—an alkaloid* first described by

*"This substance is a leucomaine, or physiological alkaloid, one of the natural products of living bodies."—P., D. & Co.]

Schreiner in '78, and also known as "Charcot-Neumann crystals."† This alkaloid is found in the animal economy, elsewhere than in the testicles, and in the various excretions, especially in the sputa of consumption; in all wasting diseases it is excreted in abundance, and hence there is philosophy and good sense in the suggestion to replace it. Tests made with this alkaloid have proven it to possess decided stimulating effects upon the cerebro-spinal centres, and there is a possibility after all, that it may be utilized as a remedy in exhaustion, etc. Moreover, Parke, Davis & Co. have prepared a salt of the alkaloid, the "hydrochlorate of spermine" for hypodermic use, and we would not be surprised shortly to see it on the market, and in more or less general use.

But as Jenner, who became immortal by the discovery that lymph from vessicles of kine-pox, introduced into the human system would forever protect from small-pox, had not the faintest conception of its rationale, but announced it is a clinical fact, and we all practiced it empirically for years, so Brown-Sequard in all probability never dreamed of the presence of an alkaloid in his "juice," which, when isolated, is capable of producing the symptoms claimed to have been observed by him; and as it remained for Klebbs, Kock and Pasteur, by their discoveries of micro-organism, to make clear the modus operandi of vaccination, so it has fallen to the lot of Parke, Davis & Co. to show up the true inwardness of the so-called "elixir vitæ," and to invest it with deep scientific interest. The Age, a medical periodical of Detroit, contains a long article on the subject, being a report of the isolation and preparation above referred to, by the chemist in the scientific department of this great establishment.

Hence our prediction (?) in last issue may yet be verified, i. e., that the "Brown-Sequard Elixir" (its active principle) may yet be offered to the public as a "sure cure," etc., and physicians will be carrying around in their hypodermic cases little pellets or tablets of the "hydrochlorate of spermine," and injecting it into the arms of divers and sundry old men and young men with as much confidence as they now inject the hydrochlorate of cocaine,

† Phosphate of spermine.

for instance; why not? One of the enterprising and intelligent young physicians of Austin has already written to Parke, Davis & Co., for a small supply of the salt, and will experiment with it, and report results to the JOURNAL.

It is right funny; the Age in arguing for the Brown-Sequard Elixir, says: "Historical record shows that the tests have been venerated for many centuries as the seat of the life-giving principle." Dr. Merriman asks us to suggest that their "juice" wasn't used hypodermically nor injected into the arm.

