# Errata

**Published:** 1889-09

**Authors:** 


ERRATA.—Page 99, editorial, for "eurdite" read "erudite." On page 101, 6th line, for "tests" read "testes." On page 100, read "vesicles" for "vessicles."

